# Graph-Based and Multi-Stage Constraints for Hand–Object Reconstruction

**DOI:** 10.3390/s26020535

**Published:** 2026-01-13

**Authors:** Wenrun Wang, Jianwu Dang, Yangping Wang, Hui Yu

**Affiliations:** 1 College of Electronics and Information Engineering, Lanzhou Jiaotong University, Lanzhou 730070, China; wangwenrun@mail.lzjtu.cn (W.W.); wangyangping@mail.lzjtu.cn (Y.W.); 11250673@stu.lzjtu.edu.cn (H.Y.); 2 National Virtual Simulation Experimental Teaching Center for Rail Transit Information and Control, Lanzhou Jiaotong University, Lanzhou 730070, China

**Keywords:** graph convolution, RGB-D adaptive fusion, physical perception, mutual attention, implicit surfaces, hand–object reconstruction

## Abstract

Reconstructing hand and object shapes from a single view during interaction remains challenging due to severe mutual occlusion and the need for high physical plausibility. To address this, we propose a novel framework for hand–object interaction reconstruction based on holistic, multi-stage collaborative optimization. Unlike methods that process hands and objects independently or apply constraints as late-stage post-processing, our model progressively enforces physical consistency and geometric accuracy throughout the entire reconstruction pipeline. Our network takes an RGB-D image as input. An adaptive feature fusion module first combines color and depth information to improve robustness against sensing uncertainties. We then introduce structural priors for 2D pose estimation and leverage texture cues to refine depth-based 3D pose initialization. Central to our approach is the iterative application of a dense mutual attention mechanism during sparse-to-dense mesh recovery, which dynamically captures interaction dependencies while refining geometry. Finally, we use a Signed Distance Function (SDF) representation explicitly designed for contact surfaces to prevent interpenetration and ensure physically plausible results. Through comprehensive experiments, our method demonstrates significant improvements on the challenging ObMan and DexYCB benchmarks, outperforming state-of-the-art techniques. Specifically, on the ObMan dataset, our approach achieves hand CD_h_ and object CD_o_ metrics of 0.077 cm^2^ and 0.483 cm^2^, respectively. Similarly, on the DexYCB dataset, it attains hand CD_h_ and object CD_o_ values of 0.251 cm^2^ and 1.127 cm^2^, respectively.

## 1. Introduction

Our hands function as the key interface for engaging with physical objects in everyday environments. The information-transmission capabilities and flexible operation of human hands are what make them so powerful. Understanding human hand actions and integrating hand operation functions into intelligent interaction scenarios is highly significant. Specifically, high-precision reconstruction of the shapes of hands and interactive objects not only boosts the realism of virtual scene interactions but also facilitates the collection of data on hand–object surface contact, enabling more rational grasping actions. Consequently, reconstructing the 3D shape of the hand–object is crucial for numerous applications, including robotic imitation learning, human–computer interfaces, fine-grained action analysis, and virtual and augmented reality.

Over the past few years, the domain of hand–object perception has garnered significant attention, particularly in areas like gesture recognition and dense reconstruction. Substantial progress has been achieved in developing algorithms for estimating hand–object poses [1,2,3,4,5,6]; much of this research has focused on sparse keypoint detection, which falls short in understanding hand–object contact dynamics. To prevent unrealistic poses, a deeper exploration of the interaction between hands and objects is essential. Consequently, there has been a shift from estimating skeletal structures to recovering the full pose and surface geometry of hands and objects. Previous studies have explored reconstruction using multi-view images [7], RGB images [8,9,10,11], depth maps [12], or point clouds [13,14]. Nevertheless, the reconstruction of hand and object shapes remains a largely underexplored domain. One major hurdle is the complexity of data collection, which often requires specialized equipment, limiting its practical applicability. Additionally, reconstructing the shapes of the hand–object simultaneously presents notable difficulties, primarily because of mutual occlusion and the wide variety of interaction patterns. Approaches that fail to address these issues often result in physically implausible outcomes, such as interpenetration or lack of contact.

Reconstructing hand–object interactions presents significant challenges, but the physics of contact also offers a simplifying factor by constraining the range of plausible configurations. Graph convolutional networks (GCNs) are particularly effective in capturing the kinematic constraints between joints and the relationships among mesh vertices of both hands and objects. Notably, poses and meshes can be viewed as graph-based representations of the hand–object system, differing in their level of detail. Sparse keypoint locations of the hand–object interaction serve as a robust prior for Signed Distance Function (SDF) estimation. To tackle this issue, we propose an approach utilizing dynamic sparse-to-dense GCNs to simultaneously reconstruct hand and object geometries. Starting from a sparse 2D pose representation, the network progressively refines the graph, increasing the number of nodes across multiple GCN layers until it achieves a dense mesh representation of the hand–object. This approach ensures the final output accurately captures the deformed mesh vertices of the interacting entities.

In our work, we tackle the problem of reconstructing hand–object interactions from a single RGB-D input by introducing a novel end-to-end framework based on a full-process graph convolution network with multi-stage constraints. Our approach progressively refines the reconstruction from sparse 2D/3D keypoints to dense, physically plausible meshes. The RGB-D input is first processed through an adaptive fusion module to extract robust multi-modal features. Structural priors are then incorporated to improve 2D pose estimation, while texture cues are leveraged to refine depth estimates for 3D pose initialization. At the core of our method, we iteratively recover dense meshes using graph convolutions, during which a dense mutual attention mechanism is applied repeatedly to dynamically model interaction patterns. Finally, we adopt an SDF-based implicit representation tailored for contact surfaces to effectively prevent interpenetration.

The primary contributions of our work are outlined below:

(1) We propose a holistic framework for hand–object reconstruction that integrates multi-stage constraints into a full-process graph convolution network, ensuring end-to-end physical plausibility from sparse keypoints to dense mesh recovery;
(2) We introduce an adaptive RGB-D fusion module and embed structural priors into 2D/3D pose estimation, substantially improving keypoint accuracy under challenging occlusion conditions;
(3) We develop a dense mutual attention mechanism that operates iteratively during mesh refinement to capture fine-grained interaction dependencies, combined with a specialized SDF representation for contact surfaces to ensure non-penetrative and geometrically consistent results;
(4) Extensive experiments on ObMan and DexYCB benchmarks show that our method achieves state-of-the-art performance in both reconstruction accuracy and physical realism.

## 2. Related Works

### 2.1. Hand–Object Joint Pose Estimation

Due to the explosive growth of deep learning, learning-based approaches for estimating hand–object keypoints have gained significant attention and achieved notable breakthroughs [1,2,3,4]. Tekin et al. [1] proposed a novel network capable of handling four essential tasks concurrently—3D pose estimation of hands and objects, object recognition, and activity classification—solely based on RGB images. Huang et al. [4] introduced a non-autoregressive Transformer-based network, named HOT-Net, which is used to estimate 3D keypoints, model hand–object correlations, and enhance physical plausibility through a pose constraint mechanism. Kuang et al. [3] utilized prior conditions to learn image background information for hand–object occlusion, offering tailored feature images to the context, hand, and object decoder layers to simplify layer separation and study characteristics.

Methods of estimating hand–object poses using graph convolutional neural networks (GCNs) [15] have gained significant attention [2,5,6], primarily due to their ability to explicitly model topological structures and interaction relationships through graph representations. Here, 21 hand joints and eight object corners are represented as nodes, while skeletal and corner connections form edges. Doosti et al. [5] proposed HOPE-Net, a lightweight framework used to estimate the 2D and 3D poses of the hand–object in real-time. It employs two adaptive GCNs in cascade: the first predicts 2D poses, while the second converts them into 3D. Zhuang et al. [2] introduced a context-aware GCN to jointly learn geometric priors and interaction information. It predicts 2D keypoints from RGB images, estimates initial 3D poses using separate subgraphs for hands and objects, and merges them into an interaction graph to refine results. Hoang et al. [6] jointly learned texture and geometric features from depth and RGB images. Using an adaptive feature-based deep Hough voting strategy and a GCN, they modeled dynamic dependencies in hand–object interactions to achieve joint 3D pose estimation. Unlike methods focusing on skeleton prediction, our work emphasizes generating dense hand meshes to better infer object interactions, delivering more intricate and detailed representations tailored for practical applications in real-world scenarios.

### 2.2. Hand–Object Shape Reconstruction

Over the past five years, advancements in hand–object reconstruction research have been notable, primarily divided into two methodological approaches: parametric mesh models [8,16,17,18] and learned representations [9,10,11,13,19,20,21,22,23,24]. Parametric mesh models are particularly favored for their extensive prior knowledge and their efficacy in predicting coherent shapes amidst challenges like occlusion, low resolution, and noise. A standout example in this category is the MANO model [25], a sophisticated parametric hand model developed from comprehensive 3D scans of human hands. MANO incorporates significant prior information, facilitating the generation of highly realistic hand meshes. Hasson et al. [16] pioneered a learnable framework based on AtlasNet [26] that uses only RGB images to simultaneously reconstruct both hand and object shapes, representing a parametric model-based approach. Despite this innovation, their approach is constrained to reconstructing only basic objects and suffers from restricted accuracy in reconstruction outcomes. Several methods [8,27,28] assume the availability of the actual 3D object model during testing as a constraint, focusing solely on predicting the object’s 6D pose to improve reconstruction results. Generating hand meshes from estimated MANO parameters yields high-quality results, but the limited resolution of the parameterized mesh hinders its ability to refine details and resolve mutual penetration between the hand and interacting objects.

To address above issues, researchers have increasingly adopted modeling methods based on learned representations, which excel in improving shape reconstruction accuracy and mitigating hand–object penetration. A key innovation is the use of implicit surface functions, particularly the Signed Distance Function (SDF) [29], which represents volumetric information by defining the distance of any point to the nearest surface, with the sign indicating its position relative to the object. This approach enables precise modeling of contact areas and mutual embedding states, facilitating the reconstruction of physically plausible mesh structures for both the hand and interacting objects [9,13,22,30]. Karunratanakul et al. [13] pioneered the use of a unified SDF to represent both the hand and the object, showcasing its capability to effectively model their interaction. Chen et al. [9] integrate parametric mesh models with SDF for joint hand–object reconstruction. They enhance learning efficiency for the hand and interactive object by separating shape and pose estimation, aligning SDF expressions with estimated poses to improve shape accuracy. Chen et al. [30] introduce a geometry-driven SDF approach used for hand–object reconstruction. It estimates poses based on kinematic chains, extracts kinematic and local visual features using geometry, and predicts signed distances. Liu et al. [31] introduce a coarse-to-fine SDF network, utilizing RGB-D modality’s perceptual strengths in both visual and geometric domains to progressively simulate implicit information, which is used for the hand–object reconstruction. Liu et al. [23] present a framework integrating implicit representations with richer prior information, thoroughly exploring additional priors and visual cues. Zhang et al. [11] propose a network architecture for the estimation of hand and interacting object pose and shape reconstruction based on monocular RGB images. By introducing multi-scale mechanisms, feature interaction, and segmentation supervision, they improved the performance of hand–object reconstruction. Woo et al. [24] introduce a refinement method grounded in graphs. This approach integrates an interaction-aware graph-attention mechanism to address hand–object interactions. By means of edges, connections are forged between nodes that are closely correlated. These linkages are set up both within single graphs and across distinct graphs.

The method introduced in this paper offers notable advantages over conventional hand–object interaction reconstruction techniques. Existing approaches [9,13,16,23,30,31,32,33,34,35], which primarily depend on end-to-end parametric model prediction or implicit SDF optimization rooted in global pose estimation, often suffer from several limitations—including sensitivity to unimodal inputs, accumulation of errors in initial pose estimation, and a general absence of physical constraints. To address these issues, we propose an innovative “sparse-to-dense” two-stage reconstruction framework. In the first stage, robust and accurate sparse hand–object pose estimation is achieved through the integration of multi-modal data and physical priors, supported by a dense interactive attention mechanism. Subsequently, dense meshes are incrementally reconstructed from these sparse correspondences. A key innovation is the incorporation of the Signed Distance Function (SDF) as a physical constraint to optimize contact regions and mitigate penetrations, thereby enhancing both the geometric accuracy and physical plausibility of the reconstructed results. This approach effectively overcomes the typical drawbacks of prior methods and enables more reliable and finely detailed reconstruction of hand–object interactions.

### 2.3. Attention-Based Methods

Attention mechanisms [36] have demonstrated significant success in estimating human body [37,38] and hand or object poses [39], excelling at capturing long-range dependencies and integrating characteristics of the target area. Leveraging their ability to model complex relationships, recent studies [10,20,40] have introduced attention-based approaches for enhanced performance. Hampali et al. [40] introduce an approach that learns attention using a sparse joint representation of the hand–object. Closely related to our work, it employs cross-attention to model hand–object interactions. Tse et al. [20] introduce an attention-guided GCN designed to efficiently integrate node features into the hand or object graphs. Their method employs self-attention to model intra-entity reliance for the hand–object, with interactions captured through the exchange of global features. Aboukahadra et al. [21] introduce THOR-Net, a framework that integrates GCNs and Transformers, only using a single RGB image to accurately model dual hands and an object shape. This framework operates through a two-step sequence: initial feature extraction and subsequent reconstruction. Existing methods primarily focus on modeling sparse interactions among a predefined collection of hand–object joints or features, overlooking the fact that hand–object interactions occur across physical surface regions. In contrast, Wang et al. [10] introduce a mutual attention mechanism that captures dependencies between every hand and object vertex. This approach enables each node in one graph to aggregate features from the other graph through an attention-based process, offering a more comprehensive representation of interactions.

Drawing inspiration from [10], this paper proposes a dense mutual interactive attention mechanism integrated at every stage of the gradual restoration process, transforming a sparse 3D graph structure into a detailed hand–object dense grid. Unlike [10], which refines the reconstructed model only after generating hand and object meshes, our approach continuously optimizes attention throughout the step-by-step restoration process. This refinement significantly enhances the accuracy of the mesh model reconstruction and the representation of interactive contact surfaces.

## 3. Method

**Figure 1:**
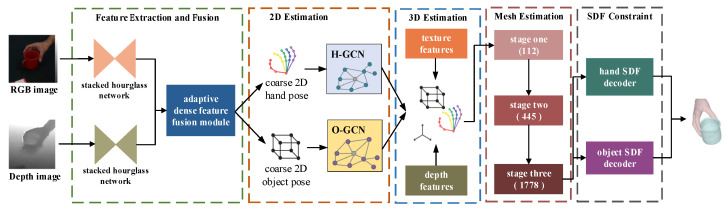
Overall structure of the network. The proposed method incorporates several essential elements: feature extraction and fusion, 2D and 3D sparse node estimation, dense mesh estimation, and refinement of the hand–object contact surface.

Accurate reconstruction of hand–object interactions is highly challenging, mainly due to severe mutual occlusion and the limited ability of existing methods to capture complex shape dependencies. This often results in low-fidelity reconstructions with physically implausible artifacts—especially surface penetration in contact regions. To address these issues, we propose a novel reconstruction framework that synergistically integrates graph convolution with multi-stage constraints. As shown in Figure 1, our model takes an RGB-D image as input and progressively reconstructs the scene through three dedicated stages: sparse node estimation, dense mesh recovery, and contact surface refinement. In the first stage, sparse node estimation accurately localizes 2D and 3D keypoints using adaptive feature fusion and a structure-aware perception module. The subsequent dense mesh recovery stage employs a coarse-to-fine strategy, combining upsampling, graph convolution, and a dense mutual attention mechanism to iteratively reconstruct complete mesh vertices. Finally, the contact surface refinement stage utilizes a Signed Distance Function (SDF) to explicitly model the interaction interface, effectively eliminating interpenetration and ensuring physical plausibility.

### 3.1. Hand–Object Sparse Node Estimation

This section details the implementation of three modules: feature extraction and adaptive fusion, 2D pose estimation with physical optimization, and 3D pose regression. Leveraging RGB-D image features and the inherent topology of hands and objects, our two-stage approach—first estimating 2D pose, then regressing 3D pose—ensures higher accuracy in sparse node estimation, enabling precise and physically plausible reconstruction of hand and object shapes.

#### 3.1.1. Feature Extraction and Adaptive Fusion

**Figure 2:**
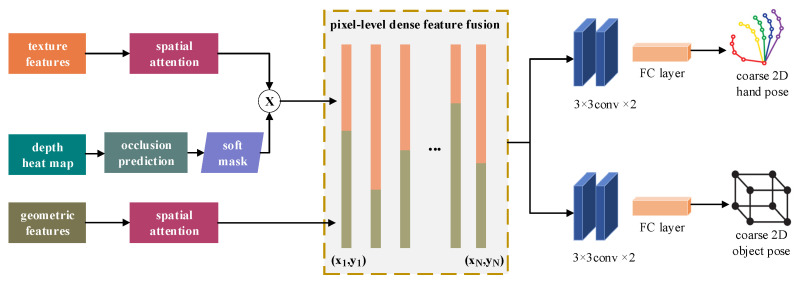
Architecture of the adaptive feature fusion network.

Existing mainstream visual sensors, such as RGB and depth cameras, face perspective limitations, making it challenging to capture all hand joint points from a single viewpoint. However, multi-modal data fusion methods significantly reduce the complexity of estimating hand and interactive object poses under single-perspective conditions. Therefore, this work takes monocular RGB-D as input and employs two structurally identical stacked hourglass networks [41] to separately extract geometric features from depth images and texture features from RGB images. Since RGB-D contains complementary information for interactive pose estimation, the texture and geometric features offer distinct advantages in accurately estimating 2D poses, especially under varying degrees of occlusion between the hand and the interactive object. The key to enhancing pose estimation accuracy lies in the effective fusion of these heterogeneous features [42]. To enhance 2D sparse node estimation, this work introduces an adaptive feature fusion framework following the feature extraction module, and the architecture of the adaptive feature fusion network is depicted in Figure 2. This network performs pixel-level discriminative dense fusion of heterogeneous features, achieving greater accuracy.

We represent the extracted texture features and geometric features as $F_{rgb}\in {\mathbb{R}}^{H\times W\times 256}$ and $F_{depth}\in {\mathbb{R}}^{H\times W\times 256}$, respectively. To suppress background interference, a spatial attention mechanism processes both modalities before pixel-level dense fusion, focusing on task-relevant regions within the global image. This yields spatial attention weight matrices $M_{rgb}^{H\times W\times 1}$ and $M_{depth}^{H\times W\times 1}$, respectively. The calculation process is formulated as follows:

(1) $$\begin{array}{l} M_{rgb}^{H\times W\times 1}=\sigma \left(conv_{7\times 7}\left(avgpool\left(F_{rgb}^{H\times W\times 256}\right),maxpool\left(F_{rgb}^{H\times W\times 256}\right)\right)\right)\\ M_{depth}^{H\times W\times 1}=\sigma \left(conv_{7\times 7}\left(avgpool\left(F_{depth}^{H\times W\times 256}\right),maxpool\left(F_{depth}^{H\times W\times 256}\right)\right)\right) \end{array}$$

where σ denotes the activation function, $conv_{7\times 7}$ represents the 7 × 7 convolution kernel, and $avgpool\left(\;\right)$ and $maxpool\left(\;\right)$ correspond to average and max pooling, respectively. Subsequently, to achieve adaptive refinement, the weight matrix and the input features are fused, producing the final output features $G_{rgb}^{H\times W\times 256}$ and $G_{depth}^{H\times W\times 256}$. The calculation process is formulated as follows:

(2) $$\left(G_{rgb}^{H\times W\times 256},G_{depth}^{H\times W\times 256}\right)=\left(\left(M_{rgb}^{H\times W\times 1}⊗F_{rgb}^{H\times W\times 256}\right),\left(M_{depth}^{H\times W\times 1}⊗F_{depth}^{H\times W\times 256}\right)\right)$$

Building on insights from [20,27], when hand occlusion by the operating object exceeds a threshold (fewer than 16 visible joints), the significant information loss in RGB images drastically increases hand estimation errors, making joint estimation with depth maps ineffective. To mitigate this, we introduce soft masking on RGB images before pixel-level dense feature fusion, leveraging depth heatmaps for occlusion prediction. Occlusion is detected by comparing the depth values of hand joints to their true z-values. If the difference surpasses the preset threshold (30 mm), the joint is classified as occluded. Severe occlusion (more than five occluded joints) reduces the soft mask weight to 0, suppressing RGB features; otherwise, the weight approaches 1, preserving RGB features for pose estimation. The soft mask is multiplied with the texture feature branch to generate feature map ${\hat{G}}_{rgb}\in {\mathbb{R}}^{H\times W\times 256}$, which is then fused with geometric features to produce output feature $F_{fusion}^{H\times W\times 256}$. The calculation process is formulated as follows:

(3) $$F_{fusion}^{H\times W\times 256}=DenseFusion\left({\hat{G}}_{rgb},G_{depth}\right)$$

#### 3.1.2. Two-Dimensional Pose Estimation and Physical Optimization

As illustrated in Figure 2, the adaptive fusion features from the previous stage are processed through two 3 × 3 convolution layers and a fully connected layer branch, which are utilized to estimate the hand and object coarse 2D poses, respectively. To represent the hand and object, we employ 21 keypoints for the hand and the 8 corner points of a bounding box for the object. It is widely recognized that the tree-like hinge structure of the hand skeleton imposes inherent geometric constraints in both 2D projection and 3D space. Additionally, the hand plays a dominant role in the hand–object interactive scenario, and precise estimation of hand posture significantly influences the object pose. Therefore, we propose learning the nonlinear mapping relationship between these elements. Before 3D pose regression, we construct a graph structure that integrates hand joints and object corners for physical feature learning.

**Figure 3:**
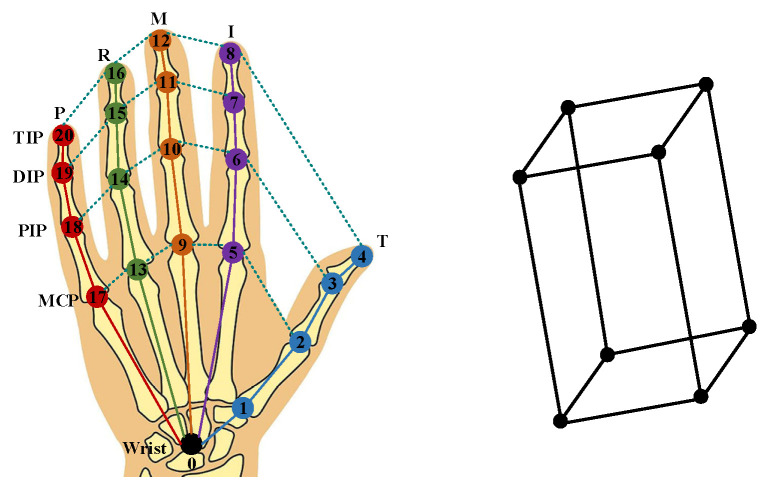
Physical structures of hands and objects. We utilize 21 keypoints for the hand and the 8 corner points of a bounding box for the object. The hand skeletal landmarks—specifically TIP (Distal Phalanx), DIP (Intermediate Phalanx), PIP (Proximal Phalanx), and MCP (Metacarpophalangeal Joint)—are used for point localization. The fingers are labeled as T (Thumb), I (Index), M (Middle), R (Ring), and P (Pinky), while numbers 0–20 denote the sequence of the 21 hand keypoints.

As shown in Figure 3, our method utilizes two dedicated subgraph networks to explicitly model the hand and object’s physical structures, significantly improving the physical plausibility of the initial coarse 2D pose estimation. The overall architecture integrates these kinematic representations through a three-layer graph convolutional network (GCN). Explicit Anatomical Connections (Hand): The hand’s skeletal topology defines inherent connectivity between joints—for example, metacarpophalangeal (MCP) joints are linked to proximal phalanges, which subsequently connect to distal phalanges. These anatomical relationships are encoded as a predefined adjacency matrix in the graph, where edges correspond to biomechanically valid connections. This fixed matrix, grounded in anatomical knowledge, preserves the structural integrity of the hand throughout training. Implicit Kinematic Constraints (Hand): In contrast to explicit connections, kinematic constraints—such as joint angle limits and intersegment dependencies—are learned automatically from data. The model constructs a data-driven adjacency matrix that captures statistical relationships between joints (e.g., synergies between finger flexion and thumb rotation). This adaptive matrix is optimized during training to emulate realistic hand movement dynamics, enhancing the model’s ability to generalize to novel poses. Object Geometric Structure: For objects, the 3D bounding box is represented by its eight corner points, forming a rigid rectangular prism. The object subgraph employs a fixed adjacency matrix derived from the box’s geometric topology, reflecting the assumption that objects are rigid and their structure remains constant.

#### 3.1.3. Three-Dimensional Pose Regression

**Figure 4:**
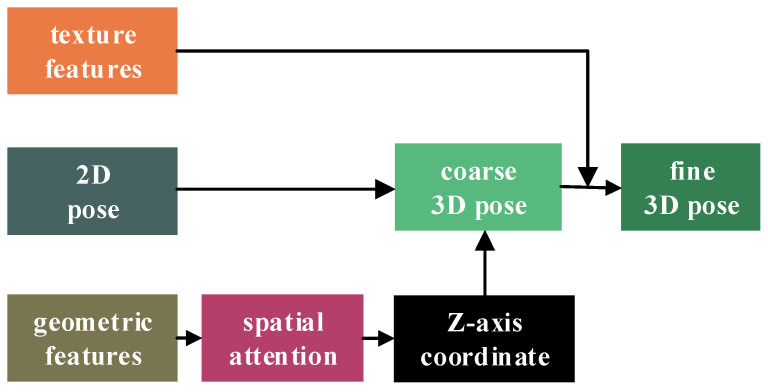
Network of 3D pose regression.

During the 3D pose regression process, depth ambiguity in RGB images is a critical consideration. Unlike RGB images, depth images directly encode the Euclidean distance from scene points to the camera’s optical center, establishing a linear mapping relationship with the z-axis coordinates of joint and corner points. Consequently, the initial z-axis coordinates of 3D hand joints and object corner points are exclusively regressed using the depth map. The architecture of the 3D pose regression network is depicted in Figure 4. Initially, the extracted geometric features are refined through spatial attention mechanisms to suppress background information, leading to enhanced z-axis coordinates of the hand–object. Subsequently, the optimized 2D coordinates derived from optimization processes are combined with the z-axis regressed from the depth map, yielding a preliminary 3D pose. Moreover, depth images are often susceptible to resolution limitations and noise interference, which can compromise the accuracy of depth values, particularly at the edge structures of the hand and interacting objects. In contrast, texture features provide a clearer distinction between the hand and interacting objects, enabling more precise identification of their shapes and poses. Therefore, we leverage texture features to optimize and constrain the initially obtained coarse 3D pose. The texture features are fused and processed alongside the coarse 3D pose by employing a multi-layer perceptron (MLP), which further refines and corrects the 3D coordinates of the hand–object.

### 3.2. Hand–Object Dense Meshes Estimation

**Figure 5:**
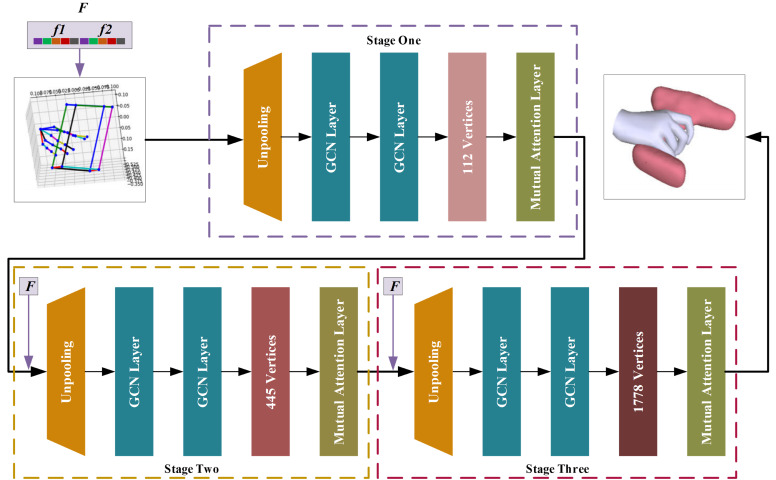
Network of dense meshes estimation. This network consists of three stages, and the characteristics for each graph node of the hand–object are initialized across the three distinct stages by leveraging the multi-modal fusion features *F*.

This section presents a novel network designed to generate dense meshes of hand–object based on a provided 3D pose. As depicted in Figure 5, the 3D nodes of the graph are enriched with a multi-modal feature vector F, which significantly boosts mesh reconstruction accuracy. The graph’s node count evolves through three sequential stages, starting at 29 and scaling up to 112, 445, and ultimately 1778 vertices. To better capture the interplay between graphs, a novel mutual attention layer is introduced, enabling refined feature aggregation across hand and object graphs.

#### 3.2.1. Hand–Object Graph Reconstruction

Inspired by [10,19,43], the characteristics for each graph node of the hand–object are initialized across the three distinct stages by leveraging the multi-modal fusion features F. With such detailed information embedded in every node, the GCNs are capable of producing highly accurate and refined representations of hands and objects. Using the initial 3D sparse graph reconstruction as an example, the pixel coordinates of the *n*-th node $V_{n}={\left[u_{n},v_{n}\right]}^{T}$ in the sparse 3D mesh are utilized to spatially sample local features from the image features $\left\{F_{\left(i\right)}\right\}$ through a bilinear interpolation operation $f_{b}\left(\cdot \right)$. Simultaneously, the final image features are fused to derive a global feature that encapsulates the overall structural information for the hand–object meshes. We used $H_{n}$ to represent the initial reconstructed node features, formed by concatenating these local and global features:

(4) $$H_{n}=f_{b}\left(\left\{F_{\left(i\right)}\left(V_{n}\right)\right\}i\in x\right)⊕f_{g}\left(F\right)$$

where $H_{n}\in {\mathbb{R}}^{k\times 3}\left(k=29,112,445\right)$, x represents a collection of layer indices used for sampling feature maps, $f_{g}\left(\cdot \right)$ serves as a module for integrating global features, and ⊕ signifies the operation of concatenation.

Following the reconstruction of node features, they are subsequently refined through graph convolutional layers. This feature-refinement process can be described as follows:

(5) $$H_{n}^{\prime }=MLPs\left(H_{n}+\sum_{i\in N_{n}}H_{i}\right)$$

where *MLPs* denotes several sequential multi-layer perceptrons and $N_{n}$ represents the indices of neighboring nodes connected to the *n*-th node. Essentially, the GCNs leverage the topological relationships within the mesh model, enabling them to effectively capture and model dependencies within the graph structure.

#### 3.2.2. Hand–Object Mesh Estimation

As illustrated in Figure 5, the network progressively reconstructs the vertices of the hand–object meshes across three stages. Each stage begins with an unpooling layer, subsequently operated by dual GCN layers to achieve a refined mesh. To enhance refinement, a dense mutual attention mechanism is applied to each stage’s output before proceeding to the next, effectively capturing hand–object interactions. This mechanism fuses complementary graph features into each node, optimizing the graph representation. Specifically, three 1D convolutional layers are applied to the hand graph’s node features to generate the query ($Q^{h}\in {\mathbb{R}}^{k^{h}\times F}$), key ($K^{h}\in {\mathbb{R}}^{k^{h}\times F}$), and value ($V^{h}\in {\mathbb{R}}^{k^{h}\times F}$) matrices, with each row representing a specific node’s corresponding feature. Similarly, the same process is performed on the object graph to produce its $Q^{o}\in {\mathbb{R}}^{k^{o}\times F}$, $K^{o}\in {\mathbb{R}}^{k^{o}\times F}$, and $V^{o}\in {\mathbb{R}}^{k^{o}\times F}$ matrices. The object-to-hand attention is then computed by aligning the hand graph’s queries with the object graph’s keys [36]. This enables the aggregation of object node features, weighted by the derived attention scores, as follows:

(6) $$H^{o\to h}=softmax\left(\frac{Q^{h}K^{o^{T}}}{\sqrt{F}}\right)V^{o}$$

where $V^{o\to h}\in {\mathbb{R}}^{k^{h}\times F}$ denotes the combined features extracted from the object graph.

Similarly, the hand-to-object attention can be calculated as follows:

(7) $$H^{h\to o}=softmax\left(\frac{Q^{o}K^{h^{T}}}{\sqrt{F}}\right)V^{h}$$

where $V^{h\to o}\in {\mathbb{R}}^{k^{o}\times F}$ denotes the combined features extracted from the hand graph.

Ultimately, we merge the aggregated feature with the original feature for every node as follows:

(8) $${\tilde{H}}_{n}=f_{v}^{h}\left(H_{n}^{\prime }⊕H^{o\to h}\right)⊕f_{v}^{o}\left(H_{n}^{\prime }⊕H^{h\to o}\right)$$

where ${\tilde{H}}_{n}$ represents the enhanced node feature produced as the output of each block, while $f_{v}^{h}\left(\cdot \right)$ and $f_{v}^{o}\left(\cdot \right)$ function as separate fusion components.

Table 1 details the parameter configuration for each stage of hand–object mesh estimation. The model takes an initial interaction graph with 29 nodes—comprising 21 hand joints and 8 object bounding box corners—as input, with its graph topology defined by physical kinematic constraints. The network progressively refines the mesh over three stages, where each stage’s graph topology is synchronized with the corresponding mesh simplification or subdivision, maintaining geometric consistency without dynamic recalculation. Upsampling is performed using predefined parent–child node mappings along with bilinear interpolation, enhanced by distance weighting and local normal constraints for feature propagation. The system utilizes approximately 9.59 million parameters and requires around 3.29 × 10^9^ FLOPs, with most resources allocated to the final high-resolution coordinate regression stage.

### 3.3. Hand–Object Surface Constraint

**Figure 6:**
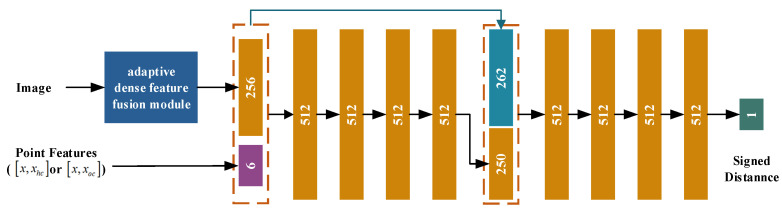
Network architecture used for hand and object SDF decoders.

Building on established methodologies [9,13,29], we employ neural networks to model SDF for the hand–object, which can facilitate an explicit and computationally efficient representation of the intricate dynamics, encompassing both contact and interpenetration phenomena for the hand–object. More precisely, the hand–object contact manifold is mathematically defined by $C=\left\{x|SDF\left(x\right)=0\;\mathrm{for}\;x\in {\mathbb{R}}^{3}\right\}$, which captures the spatial and temporal characteristics of surface interactions. Simultaneously, the volume of hand–object interpenetration is precisely quantified by $I=\left\{x|SDF\left(x\right)<0\;\mathrm{for}\;x\in {\mathbb{R}}^{3}\right\}$, providing a measure of the extent to which the hand and object overlap in 3D space. As depicted in Figure 6, the hand and object SDF decoder processes 256-dimensional image features F and 6-dimensional point features. For a given 3D point x, the hand SDF decoder computes its signed distance to the hand surface, while the object SDF decoder calculates the distance to the object surface.

To compute the hand Signed Distance Field (SDF), the process begins by extracting the global rotation and its rotation center from the hand mesh parameters. These parameters are used to map the original 3D point x to a corresponding point $x_{hc}$ in the “canonical hand pose”—a pose with zero global rotation—through an inverse rotation matrix operation. This step effectively removes the influence of hand rotation on shape modeling, ensuring a more accurate representation of the hand’s intrinsic geometry. Next, the original 3D point and its canonical counterpart are combined to form enriched point features. These features, along with multi-modal inputs such as 256-dimensional image features F and 6-dimensional point features, are fed into the hand SDF decoder. The decoder then predicts the signed distance from the input point to the hand surface, providing a precise measure of spatial proximity.

The object SDF calculation follows a similar logic: extract the translation vector from object pose estimation, map the original point x to a corresponding point $x_{oc}$ in the “canonical object pose” (zero translation) to remove translation interference, merge the original and canonical points, input them—along with multi-modal features F—into the object SDF decoder, and output the signed distance to the object surface. During testing, the Marching Cubes algorithm [44] is employed to reconstruct the corresponding meshes.

### 3.4. Loss Function

Our approach encompasses multiple tasks, such as sparse node estimation loss $L_{sparse}$, dense meshes estimation loss $L_{dense}$, and surface constraint loss $L_{sdf}$. To enhance performance across these tasks, we employ multitask learning, which has proven more effective than training each task independently [45]. Consequently, our model is trained from end to end by minimizing the combined losses mentioned above.

(9) $$L_{total}=L_{sparse}+L_{dense}+L_{sdf}$$

Sparse node estimation loss: The loss function in this section comprises two components: the 2D and 3D pose estimation losses $L_{2D}$ and $L_{3D}$, formulated as follows:

(10) $$L_{sparse}=\lambda_{2D}L_{2D}+\lambda_{3D}L_{3D}=\sum_{i=1}^{N}{\left\|{\hat{P}}_{i}^{2D}-P_{i}^{2D}\right\|}_{2}^{2}+\sum_{j=1}^{M}{\left\|{\hat{P}}_{j}^{3D}-P_{j}^{3D}\right\|}_{2}^{2}$$

where we set $\lambda_{2D}$ and $\lambda_{3D}$ to 1, ${\hat{P}}_{i}^{2D}$ and ${\hat{P}}_{j}^{3D}$ represent the estimated values of 2D and 3D sparse nodes, and $P_{i}^{2D}$ and $P_{j}^{3D}$ denote the ground truth values of 2D and 3D sparse nodes.

Dense meshes estimation loss: Following [10], the loss function in this section comprises three components: vertex loss $L_{v}$, normal loss $L_{n}$, and edge loss $L_{e}$. We utilize an L1 loss for supervision, formulated as follows:

(11) $$\begin{array}{l} L_{dense}=\lambda_{v}L_{v}+\lambda_{n}L_{n}+\lambda_{e}L_{e}\\ \;\;\;\;\;\;\;\;\;=\lambda_{v}\left(\sum_{i}{\left\|\left|{\tilde{v}}_{i}^{h}\right|-\left|v_{i}^{h}\right|\right\|}_{1}+\sum_{j}{\left\|\left|{\tilde{v}}_{j}^{o}\right|-\left|v_{j}^{o}\right|\right\|}_{1}\right)\\ \;\;\;\;\;\;\;\;\;+\lambda_{n}\left(\sum_{i}{\left\|\left|{\tilde{n}}_{i}^{h}\right|-\left|n_{i}^{h}\right|\right\|}_{1}+\sum_{j}{\left\|\left|{\tilde{n}}_{j}^{o}\right|-\left|n_{j}^{o}\right|\right\|}_{1}\right)\\ \;\;\;\;\;\;\;\;\;\;+\lambda_{e}\left(\sum_{i}{\left\|\left|{\tilde{e}}_{i}^{h}\right|-\left|e_{i}^{h}\right|\right\|}_{1}+\sum_{j}{\left\|\left|{\tilde{e}}_{j}^{o}\right|-\left|e_{j}^{o}\right|\right\|}_{1}\right) \end{array}$$

where we set $\lambda_{v}$, $\lambda_{n}$, and $\lambda_{e}$ to 1, ${\tilde{v}}_{i}^{h}$ and $v_{i}^{h}$ represent the predicted and ground truth values of the hand mesh vertex, and ${\tilde{v}}_{j}^{o}$ and $v_{j}^{o}$ correspond to the object mesh vertex. Similarly, ${\tilde{n}}_{i}^{h}$ and $n_{i}^{h}$ denote the hand mesh edge vector, ${\tilde{n}}_{j}^{o}$ and $n_{j}^{o}$ represent the object mesh edge vector, ${\tilde{e}}_{i}^{h}$ and $e_{i}^{h}$ indicate the hand mesh edge normal, and ${\tilde{e}}_{j}^{o}$ and $e_{j}^{o}$ describe the object mesh edge normal.

Surface constraint loss: Following [10,16], the loss function in this section comprises three components: reconstruction loss $L_{rec}$, interpenetration loss $L_{ip}$, and contact loss $L_{c}$. The reconstruction loss is computed independently of the hand and the object, based on each query point x and the multi-modal feature F. Additionally, interpenetration and contact losses are incorporated during training to enhance the hand–object 3D reconstruction of the surface contact regions. The overall loss $L_{sdf}$ is defined as follows:

(12) $$\begin{array}{l} L_{sdf}=\lambda_{rec}L_{rec}+\lambda_{ip}L_{ip}+\lambda_{c}L_{c}\\ \;\;\;\;\;\;=\lambda_{rec}\left(\sum_{p\in \left\{p^{h},p^{o}\right\}}\left|c\left(f_{sdf}\left(x,F\right),\delta \right)-c\left(SDF_{p}\left(x\right),\delta \right)\right|\right)\\ \;\;\;\;\;\;\;\;+\lambda_{ip}\left(\sum_{x}max\left(-\langle 1,f_{sdf}\left(x,F\right)\rangle ,0\right)\right)\\ \;\;\;\;\;\;\;\;\;+\lambda_{c}\left(\sum_{x}min\left(\alpha {\left|f_{sdf}\left(x,F\right)\right|}^{2},1\right)\right) \end{array}$$

where we set $\lambda_{rec}$, $\lambda_{ip}$, and $\lambda_{c}$ to 1, $f_{sdf}$ serves as a surface constraint network, and $SDF_{p}\left(\cdot \right)$ represents the ground truth SDF for the component p, which can be either the hand or the object. The thresholding function $c\left(s,\delta \right)\;:=min\left(\delta ,max\left(-\delta ,s\right)\right)$ is employed to restrict the distance s within the range $\left[-\delta ,\delta \right]$, with δ uniformly set to 10 mm across all experiments. 1 represents a 2D one-vector, and $\langle \cdot ,\cdot \rangle$ is a dot product. α is a hyper-parameter, which is empirically set to 0.005. It is evident that $f_{sdf}\left(x,F\right)=0$ corresponds to the hand and the object being in contact. Hence, it disregards points associated with the predicted grasping field ${\left|f_{sdf}\left(x,F\right)\right|}^{2}\geq \frac{1}{\alpha }$, focusing solely on encouraging points marked by ${\left|f_{sdf}\left(x,F\right)\right|}^{2}<\frac{1}{\alpha }$ to serve as contact points.

## 4. Experiments

### 4.1. Datasets

**ObMan (Object Manipulation)** [16]. A large-scale synthetic image dataset dedicated to capturing hand–object interactions, it is curated from ShapeNet [46] and encompasses 21,000 hand grasp poses across 2700 diverse objects spanning 8 distinct categories. Consistent with methodologies from prior research [9,13,29,30,31], meshes with an excessive number of double-sided triangles were filtered out prior to splitting the data into training and testing sets, yielding 87,190 training samples and 6285 testing samples.

**DexYCB** [47]. Captured using multiple RGB-D cameras, the dataset features hand–object interactions across 1000 sequences, with 582,000 RGB-D frames recorded from 8 viewpoints. It includes 10 subjects grasping 20 distinct objects. Following the dataset split methodology in [30,31], samples lacking hand–object interactions are filtered out, and then the videos are resampled to a uniform 6 frames per second (fps). The result is a dataset split of 29,656 training samples and 5928 testing samples.

### 4.2. Evaluation Metrics

Our model produces structured outputs, making it insufficient to rely on a single metric for comprehensive performance assessment. To thoroughly evaluate the proposed method, we adopt multiple evaluation metrics established in prior research [9,23,31].

To comprehensively evaluate the reconstruction quality, the following metrics are employed: hand Chamfer distance (CD_h_) and F-score (F_h_@1/F_h_@5) are used to assess the accuracy of the hand mesh (cm^2^), where CD_h_ reflects the overall surface error and F-score measures completeness at specific thresholds. Similarly, object Chamfer distance (CD_o_) and F-score (F_o_@5/F_o_@10) are utilized to evaluate the quality of the object mesh (cm^2^). Additionally, hand joint error (E_h_) measures the deviation of 21 keypoints, while object center error (E_o_) quantifies the accuracy of object translation prediction (cm).

Furthermore, to evaluate the geometric plausibility of hand–object interaction, we employ the following metrics: contact ratio (C_r_) measures the proportion of samples with non-zero interpenetration between the hand and object; penetration depth (P_d_) calculates the maximum distance (cm) from hand mesh vertices to the object surface; intersection volume (I_v_) estimates the volume of the intersection (cm^3^) by voxelizing both meshes at a resolution of 0.5 cm.

### 4.3. Implementation Details

The experiments are performed on a high-performance workstation featuring an NVIDIA RTX 4090 GPU, a 10-core Xeon Platinum 8352V CPU, and 50 GB of memory, operating on Ubuntu 20.04 with Python 3.10 and PyTorch 2.4.0. GPU acceleration is enabled through CUDA 11.8 and cuDNN 8. We employ the Adam optimizer with an initial learning rate of 1 × 10^−4^, which is reduced by half at epoch 600, and train all models for 800 epochs with a batch size of 32 on both the DexYCB and ObMan datasets. During training, RGB-D images are cropped to 256 × 256 and augmented with random rotation and color jittering. Additionally, we sample 3D points and their signed distances to hand and object surfaces for SDF training. Each hand–object mesh pair is translated to center the hand root joint at the origin and scaled uniformly to fit within a unit cube. We then sample 40,000 points uniformly within the cube. For Chamfer distance computation, 30,000 points are sampled from both ground truth and reconstructed mesh surfaces. During SDF training, 1000 points (500 inside and 500 outside the mesh) are randomly sampled per hand and object. All meshes are reconstructed at a resolution of 128^3^, ensuring high-quality geometric representation.

### 4.4. Comparisons with the State-of-the-Arts

Our method’s performance in hand–object reconstruction is benchmarked against six leading techniques on the ObMan dataset, with detailed results presented in Table 2. This comparative analysis highlights the effectiveness of our approach in achieving superior reconstruction accuracy. Across multiple metrics, our method demonstrates substantial superiority, particularly in F-score at various thresholds and Chamfer distance. Notably, compared to Liu et al. [31], our approach reduces the CD_h_ and CD_o_ metrics by 0.006 cm^2^ and 0.027 cm^2^, respectively. Additionally, it achieves more precise pose estimation, lowering the hand pose error E_h_ by 0.044 cm and the object pose error E_o_ by 0.673 cm. These findings underscore the substantial progress our method has made in enhancing both reconstruction precision and pose estimation. Simultaneously, the experiments reveal that the proposed approach delivers a marked improvement in object pose estimation accuracy and dense mesh restoration, outperforming hand estimation. This highlights the critical role of hand–object physical structure optimization and the dense mutual attention mechanism in jointly estimating hand–object interactions. Moreover, precise hand pose estimation is pivotal for achieving accurate object pose estimation.

On the DexYCB benchmark, our method demonstrates a clear and consistent superiority across all critical metrics, as evidenced in Table 3, significantly surpassing the results of the previous most advanced approaches. Notably, it achieves substantial improvements, reducing errors by 0.016 cm^2^ in CD_h_ and 0.113 cm^2^ in CD_o_, which reinforces its standing as a pioneering solution in the domain. Furthermore, our approach achieves more precise pose estimation, decreasing the hand pose error E_h_ by 0.089 cm and the object pose error E_o_ by 0.353 cm. The proposed method exhibits consistent and robust accuracy when applied to real-world data, highlighting its exceptional ability to generalize effectively. These findings underscore its reliability and adaptability across diverse scenarios.

To enhance the assessment of geometric plausibility in hand–object interactions and mitigate issues such as mutual penetration or lack of contact, we adopted additional metrics: contact ratio (C_r_), penetration depth (P_d_), and intersection volume (I_v_). These metrics provide detailed insights into the interaction between hand and object meshes, emphasizing stable contact while minimizing interpenetration. Table 4 highlights that our approach outperforms in contact ratio across the DexYCB and ObMan datasets, alongside relatively low penetration depth and intersection volume. This indicates a more detailed and realistic representation of hand–object interactions. Notably, while Grassing Field [13] demonstrates lower penetration depth and intersection volume, it reconstructs hand–object contact in only 69.6% of test samples on the ObMan dataset, falling significantly lower than our method’s 96.9%. This further emphasizes the superiority of our approach in producing precise and reliable interactions, as evidenced by its ability to consistently outperform existing methods across multiple metrics. Additionally, the outstanding performance of our method on the DexYCB dataset demonstrates its robustness in managing real-world image scenarios, where challenges such as occlusions, varying lighting conditions, and complex object geometries are prevalent.

### 4.5. Ablation Study

To assess the impact of individual components in the proposed method, a comprehensive set of ablation studies was performed using the DexYCB dataset. These experiments systematically evaluated the contribution of each module by selectively removing or altering them, thereby isolating their influence on the overall performance. As detailed in Table 5, we evaluated the impact of several accuracy optimization constraint modules: the Physical Optimization Module (POM) in the sparse node estimation stage, the Mutual Attention Module (MAM) in the dense grid estimation stage, and the Surface Constraint Module (SCM) at the network’s final stage. The results demonstrate that each module contributes to improving object reconstruction accuracy, with varying degrees of enhancement. Notably, the Mutual Attention Module (MAM) has the most substantial effect, reducing CD_h_ and E_h_ metrics by 0.097 cm^2^ and 0.28 cm, respectively, and CD_o_ and E_o_ metrics by 0.653 cm^2^ and 0.432 cm, respectively. The significant enhancement in object pose estimation accuracy reveals the pivotal role of the hand in hand–object interactions, emphasizing how precise hand pose estimation directly impacts the accuracy of object pose estimation.

Additionally, to thoroughly evaluate the efficacy of our approach, we implemented a series of ablation studies, examining various input modalities—such as RGB, depth, RGB-D, and RGB-D adaptive feature fusion (RGB-D + AFF)—alongside different feature fusion techniques. These experiments aimed to assess the impact of the proposed RGB-D adaptive feature fusion method on improving hand–object reconstruction accuracy. We observed that multi-modal input images consistently outperform single-modal inputs, significantly reducing errors across all evaluation metrics, as demonstrated in Table 6. Notably, our adaptive feature fusion method significantly outperforms proportional RGB-D feature fusion, decreasing CD_h_ and E_h_ metrics by 0.017 cm^2^ and 0.129 cm, respectively, and CD_o_ and E_o_ metrics by 0.132 cm^2^ and 0.207 cm, respectively. These results highlight the advantages of adaptively integrating color and geometric features, particularly in scenarios where mutual occlusion between the hand and interactive objects varies. This demonstrates that leveraging complementary information from different modalities effectively enhances reconstruction accuracy in complex interaction scenarios.

Moreover, to reconstruct detailed hand and object meshes, we employ the Surface Constraint Module (SCM). This module processes the output dense grid by feeding it into the hand and object SDF decoders, effectively mitigating the issue of mutual penetration between hands and interacting objects. This results in more plausible and accurate hand–object reconstructions. As demonstrated in Table 7, a closer examination of the hand–object interaction metrics (C_r_, P_d_, I_v_) reveals significant improvements. Specifically, the SCM significantly enhances the realism and accuracy of hand–object interactions, as evidenced by the reconstructed hand–object being in contact in over 97.3% of test samples. Additionally, the P_d_ and I_v_ metrics are reduced by 0.19 cm and 1.51 cm^3^, respectively, further validating its effectiveness. It is important to highlight that removing the POM from the network significantly increases the degree of mutual penetration between structural components, thereby resulting in an implausible and physically inconsistent reconstruction.

### 4.6. Qualitative Results

**Figure 7:**
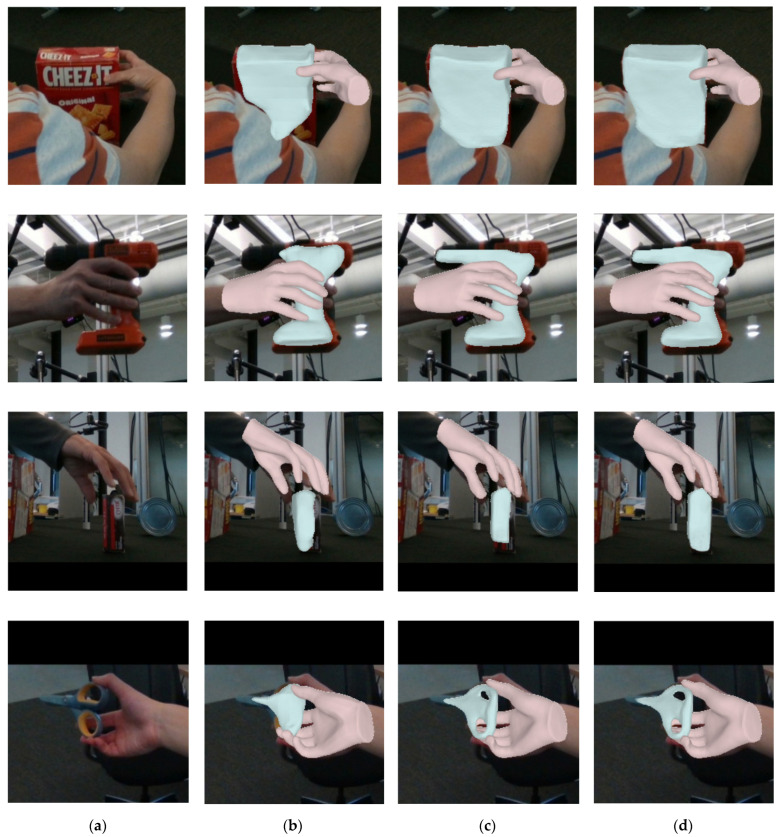
Qualitative comparison experiment between our method and other approaches on the DexYCB dataset. (**a**) Input images; (**b**) gSDF [30] method reconstruction results; (**c**) Liu et al. [31] method reconstruction results; (**d**) our method’s reconstruction results.

To assess our method’s effectiveness in hand–object reconstruction, we performed a visual analysis on the DexYCB dataset, comparing it with two leading approaches. Figure 7 showcases four interactive objects with distinct grasping postures. The findings reveal superior reconstruction quality with our technique. Although hand pose reconstruction saw limited gains, it achieved notable advancements in object detail, yielding shapes that align closely with real-world counterparts. These results underscore the method’s precision in capturing fine details and relationships, significantly reducing unrealistic mesh artifacts.

**Figure 8:**
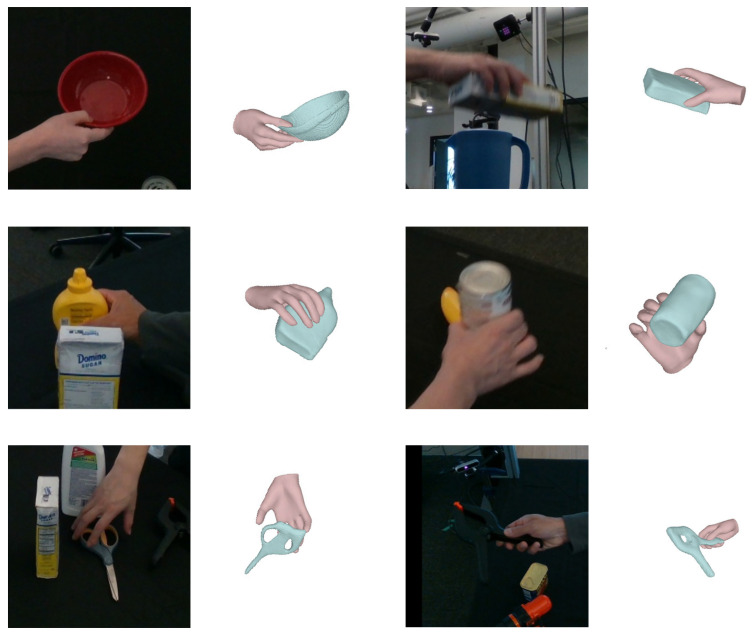
The qualitative experimental results across various scenarios on the DexYCB dataset.

Furthermore, Figure 8 presents qualitative experimental results of hand–object interaction operations across various scenes in the DexYCB dataset. The figure demonstrates that, despite varying levels of mutual occlusion and complex backgrounds, the proposed method achieves physically plausible and accurate hand–object reconstruction. This is accomplished by leveraging the complementary information from spatial and texture features, as well as the hand and object posture information learned through the multi-stage constraint mechanisms.

**Figure 9:**
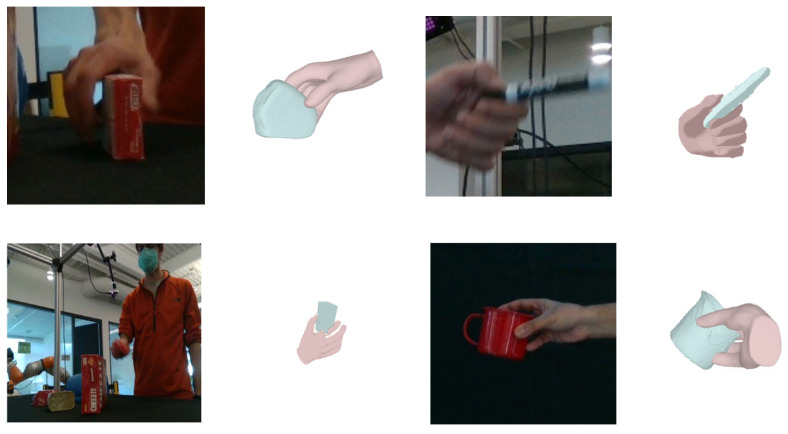
Failure cases of our method on the DexYCB dataset.

Additionally, Figure 9 illustrates failure cases of our method on the DexYCB dataset, highlighting its limitations in challenging scenarios—such as irregular object geometry, minimal object scale, ambiguous shapes due to occlusion or material properties, and strong lighting or shadow interference. These cases reveal a decline in reconstruction accuracy under severe single-view ambiguities, exposing the boundaries of our approach in handling complex geometric priors, scale variations, and environmental interference. Nonetheless, these results reinforce our argument that reconstructing hand–object interactions from a single view remains fundamentally difficult due to heavy occlusion.

### 4.7. Runtime and Computational Complexity

To evaluate the real-time performance and computational efficiency of our method, we measured both inference time and GPU memory usage. Our system processes a single image in 3.429 s and consumes 4125 MB of GPU memory. This computational overhead is a direct consequence of our architectural decisions—specifically, the multi-stage graph convolutional refinement and the SDF-based Physical Optimization Module—which are critical to producing high-fidelity, physically consistent reconstructions, the core contribution of this work. Although this precludes real-time use, the marked gains in accuracy and substantial reduction in physical interpenetration make it highly suitable for applications demanding high precision. Future efforts will focus on developing a more efficient network architecture and optimizing 3D sampling strategies to improve inference speed and facilitate practical deployment.

## 5. Discussion

The success of our method stems from key design choices that directly address prior limitations. First, by leveraging GCNs throughout the pipeline, we capitalize on the inherent graph topology of hand and object meshes. Unlike previous approaches that isolate components (e.g., pose, mesh) or rely on non-graph representations, our GCN-based framework enables a seamless transition from sparse to dense representations. This ensures global consistency, propagating information from coarse joint locations to detailed surface geometry. Additionally, our multi-stage constraint modules are pivotal in achieving physically plausible and high-precision reconstructions.

This technology holds immense potential in the realm of high-precision interaction modeling. In human–robot interaction (HRI), it can empower robots with a deeper understanding of human manipulation intent, fostering more intuitive collaboration. In virtual and augmented Reality (VR/AR), it facilitates natural hand avatar control and realistic object manipulation, significantly enhancing user immersion. Furthermore, in fields such as biomechanics and healthcare, its precise reconstruction capabilities can be leveraged for applications like rehabilitation training assessment, surgical simulation, and ergonomic design optimization.

Despite its impressive performance, the method is not without limitations. Firstly, its dependence on RGBD input restricts its applicability in scenarios where only monocular RGB cameras are available. Expanding the framework’s applicability to monocular input will be a key focus in future research, aiming to enhance its versatility and scope. Secondly, while the current physical priors effectively prevent physically implausible states, modeling continuous physical phenomena such as soft tissue deformation and complex dynamics remains an area for further exploration. Beyond addressing these input modality and modeling constraints, optimizing the network architecture itself presents a promising avenue for enhancing reconstruction fidelity, particularly under challenging conditions characterized by severe occlusions or extreme poses [48,49]. Investigating mechanisms such as pose-aware feature recalibration or dynamic graph attention may facilitate the development of more accurate and generalizable representations across the full spectrum of hand–object interactions.

## 6. Conclusions

We propose a multi-stage constraint-based framework for hand–object reconstruction that employs graph convolutional networks across the entire pipeline. By capitalizing on the inherent graph structure of hand and object poses and meshes, our approach efficiently transitions from sparse to dense representations. The integration of adaptive feature fusion, physical priors, mutual attention, and contact refinement modules enables our method to produce physically plausible and high-fidelity reconstructions. Comprehensive evaluations on the ObMan and DexYCB benchmarks show that our framework consistently outperforms state-of-the-art methods across all evaluation metrics. Specifically, it achieves a CD_h_ of 0.077 cm^2^ and a CD_o_ of 0.483 cm^2^ on ObMan, and 0.251 cm^2^/1.127 cm^2^ on DexYCB, alongside higher contact accuracy and reduced penetration.

For future work, we aim to extend the framework to more challenging and incomplete multi-modal scenarios. Motivated by recent progress in multi-view learning, we intend to investigate: Localized sparse incomplete multi-view clustering to accommodate partially available or highly sparse view inputs [50,51]; Reliable representation learning for incomplete multi-view and multi-label classification with missing labels, facilitating joint reasoning for shape reconstruction and semantic inference under incomplete data [52]. These research directions are designed to improve the robustness and generalizability of hand–object reconstruction in real-world settings, where data is often imperfect or multi-faceted.

## Figures and Tables

**Table 1:** Parameters configuration for each stage of hand–object mesh estimation. GNN represents number of graph nodes, GT represents graph topology, GCN-C (I → O) represents GCN block channels (input → output), AMS (H → O, O → H) represents attention matrix size (hand → object, object → hand), and AFD represents attention feature dimension.

Stage	GNN	GT	GCN-C (I → O)	AMS (H → O, O → H)	AFD	Params	FLOPs
One	112	Preserving hand–object key structures, with an average node degree of 6–8	10,003 → 2048	56 × 56, 56 × 56	2048	~1.89	~4.63 × 10^8^
Two	445	Preserving hand–object key structures, with an average node degree of 7–9	2048 → 1024	222 × 223, 223 × 222	1024	~2.35	~9.21 × 10^8^
Three	1778	Preserving hand–object key structures, with an average node degree of 8–10	1024 → 3	778 × 1000, 1000 × 778	3	~5.35	~1.91 × 10^9^

**Table 2:** Comparison with other methods on the ObMan dataset. ↓ indicates that a smaller value is better; ↑ indicates that a larger value is better.

Methods	CD_h_ ↓	F_h_@1 ↑	F_h_@5 ↑	CD_o_ ↓	F_o_@5 ↑	F_o_@10 ↑	E_h_ ↓	E_o_ ↓
Hasson et al. [16]	0.415	0.138	0.751	3.600	0.359	0.590	1.130	-
Grasping Field [13]	0.261	-	-	6.800	-	-	-	-
AlignSDF [9]	0.136	0.302	0.913	3.380	0.404	0.636	1.270	3.290
gSDF [30]	0.112	0.332	0.935	3.140	0.438	0.660	0.930	3.430
Liu et al. [23]	0.106	0.335	0.937	2.290	0.472	0.698	0.877	3.120
Liu et al. [31]	0.083	0.416	0.959	0.510	0.780	0.891	0.840	2.610
Woo et al. [24]	0.105	-	-	3.961	-	-	1.120	-
Ours	0.077	0.402	0.965	0.483	0.802	0.934	0.796	1.937

**Table 3:** Comparison with other methods on the DexYCB dataset. ↓ indicates that a smaller value is better; ↑ indicates that a larger value is better.

Methods	CD_h_ ↓	F_h_@1 ↑	F_h_@5 ↑	CD_o_ ↓	F_o_@5 ↑	F_o_@10 ↑	E_h_ ↓	E_o_ ↓
Hasson et al. [16]	0.537	0.115	0.647	1.940	0.383	0.642	1.670	-
Grasping Field [13]	0.364	0.154	0.764	2.060	0.392	0.660	-	-
AlignSDF [9]	0.358	0.162	0.767	1.830	0.410	0.679	1.580	1.780
gSDF [30]	0.302	0.177	0.801	1.550	0.437	0.709	1.440	1.960
Liu et al. [31]	0.267	0.185	0.823	1.240	0.488	0.764	1.010	1.380
Woo et al. [24]	0.154	-	-	5.345	-	-	1.570	-
Ours	0.251	0.194	0.842	1.127	0.506	0.794	0.921	1.027

**Table 4:** Comparison with other methods on different datasets. ↓ indicates that a smaller value is better; ↑ indicates that a larger value is better.

Datasets	ObMan			DexYCB		
	C_r_ ↑	P_d_ ↓	I_v_ ↓	C_r_ ↑	P_d_ ↓	I_v_ ↓
Hasson et al. [16]	94.8%	1.20	6.25	95.7%	1.15	9.64
Grasping Field [13]	69.6%	0.23	0.20	96.0%	0.92	6.62
AlignSDF [9]	95.5%	0.66	2.81	96.6%	1.08	8.40
gSDF [30]	89.8%	0.42	1.17	95.4%	0.94	6.55
Liu et al. [31]	96.1%	0.45	1.67	97.0%	0.81	5.36
Woo et al. [24]	-	0.69	3.50	-	0.92	5.90
ours	96.9%	0.37	1.09	97.3%	0.78	5.17

**Table 5:** Different constraint module ablation experiments on the DexYCB dataset. ↓ indicates that a smaller value is better.

Models	CD_h_ ↓	CD_o_ ↓	E_h_ ↓	E_o_ ↓
w/o POM	0.306	1.529	1.172	1.398
w/o MAM	0.348	1.780	1.201	1.459
w/o SCM	0.271	1.293	1.030	1.264
Ours	0.251	1.127	0.921	1.027

**Table 6:** Different input images ablation experiments on the DexYCB dataset. ↓ indicates that a smaller value is better.

Image	CD_h_ ↓	CD_o_ ↓	E_h_ ↓	E_o_ ↓
RGB	0.298	1.514	1.195	1.607
Depth	0.277	1.376	1.102	1.442
RGB-D	0.268	1.259	1.050	1.234
RGB-D + AFF	0.251	1.127	0.921	1.027

**Table 7:** Surface Constraint Module ablation experiments on the DexYCB dataset. ↓ indicates that a smaller value is better; ↑ indicates that a larger value is better.

Models	C_r_ ↑	P_d_ ↓	I_v_ ↓
w/o POM	94.5%	0.89	6.31
w/o SCM	93.1%	0.97	6.68
Ours	97.3%	0.78	5.17

## Data Availability

The data that support the findings of this study are available from the corresponding author upon reasonable request.

## Abbreviations

The following abbreviations are used in this manuscript:

SDF	Signed Distance Function
2D	Two-Dimensional
3D	Three-Dimensional
RGB	Red, Green, Blue
RGB-D	Red, Green, Blue-Depth
GCNs	Graph Convolutional Networks
